# Vitronectins produced by human cirrhotic liver and CCl_4_‐treated rats differ in their glycosylation pattern and tissue remodeling activity

**DOI:** 10.1002/2211-5463.12616

**Published:** 2019-03-18

**Authors:** Kimie Date, Risa Suzuki, Sachie Oda‐Tamai, Haruko Ogawa

**Affiliations:** ^1^ Human Life Innovation Institute Ochanomizu University Tokyo Japan; ^2^ Graduate School of Humanities and Sciences Ochanomizu University Tokyo Japan; ^3^ Department of Biochemistry St Marianna University School of Medicine Kawasaki Japan

**Keywords:** carbon tetrachloride, collagen, ECM: extracellular matrix, glycosylation, liver cirrhosis, vitronectin

## Abstract

Liver cirrhosis (LC) is a disease characterized by pathological accumulation and alteration of extracellular matrix (ECM) proteins; the interaction between two such proteins, collagen and vitronectin (VN), is considered to be the key to controlling ECM remodeling in liver cirrhosis. If it is possible to control the modification of oligosaccharides on VN, it may be possible to retard progression of liver cirrhosis. In this study, we examined the relationship between changes in VN glycosylation and activity related to the remodeling of hepatic tissue in human LC and a rat model of LC generated using carbon tetrachloride (CCl_4_). Plasma concentrations of VN in human LC declined to approximately two‐thirds that in normal plasma, but the ratio of active VN, which has collagen‐binding activities, increased 2.8 times in LC plasma. In contrast, purified LC‐VN exhibited similar binding activities toward type I, IV, and V collagens to those of normal VN. Lectin reactivities and carbohydrate analyses of LC‐VN revealed that branching, fucosylation, and sialylation of *N*‐glycans were higher than those of normal VN. On the other hand, the plasma level of rat CCl_4_‐VN increased and the ratio of active molecules to collagen in plasma decreased. Increased fucosylation of LC‐VN was not detected in carbohydrates of CCl_4_‐VN. The changes in rat VN due to CCl_4_ treatment did not correspond to the changes in plasma levels of human VN caused by LC, the ratio of active molecules, or carbohydrate composition, thereby indicating that CCl_4_‐treated rats are not an appropriate model for studying VNs in human LC. Glycosidase treatment of VNs supported the hypothesis that the collagen‐binding activity of VN is modulated by alterations of glycosylation during LC, which may contribute to (a) the matrix incorporation of VN and (b) tissue fibrosis.

**Abbreviations**
AAL: *Aleuria aurantia* lectin
ALT: alanine aminotransferase
AST: aspartate aminotransferase
CBB: Coomassie brilliant blue
CCl_4_: carbon tetrachloride
ConA: concanavalin A
DMB: dihydrochloride
DSA: *Datura stramonium* agglutinin
ECM: extracellular matrix
Fuc: fucose
Gal: galactose
GalNAc: *N*‐acetylgalactosamine
GlcNAc: *N*‐acetylglucosamine
HRP: horseradish peroxidase
hVN: human vitronectin
LC: liver cirrhosis
LEL: *Lycopersicum esculentum* lectin
Man: mannose
NeuAc: *N*‐acetylneuraminic acid or sialic acid
NeuGc: *N*‐glycolylneuraminic acid
PA: 2‐aminopyridine
PVDF: polyvinylidene fluoride
PVL: *Psathyrella velutina* lectin
pVN: porcine vitronectin
RCA: *Ricinus communis* agglutinin
SNA: *Sambucus nigra* bark agglutinin
TBil: total bilirubin
UEA‐I: *Ulex europaeus* agglutinin
VN: vitronectin

Vitronectin (VN) is a multifunctional adhesive glycoprotein that is present in normal plasma at high concentrations (0.2–0.4 mg·mL^−1^ in humans) 1, 2. The liver is the primary source of plasma VN, and hepatocytes have been shown to synthesize large quantities of VN 3, 4. VN regulates the blood systems related to protease cascades such as cell lysis by complement, coagulation, and fibrinolysis 5. Both inactive monomeric and active multimeric forms of VN have been identified in circulating blood 6. Most VN in normal plasma exists mainly as an inactive monomeric form that scarcely binds to its ligands, but it acquires binding activity to its ligands in a multimeric forms by treatment with denaturants such as urea *in vitro* 7. This characteristic is the basis of the VN purification method using heparin‐affinity chromatography 8. VN is also found in the extracellular matrix (ECM) of most tissues and is considered to play a role in cell adhesion, cell motility, and matrix remodeling; VN is present as an active multimeric form that interacts with various matrix ligands, such as type 1 plasminogen activator inhibitor and urokinase receptor to regulate pericellular proteolysis, various types of integrins on the cell surface, and various types of collagen 2, 4, 9.

Vitronectin is a glycoprotein. It is considered that *N*‐glycosylation of VN is critical for its functions and conformation because the consensus sequences for *N*‐glycosylation are highly preserved in mammals 10. In our previous study, the structures of *N*‐glycans of porcine and human plasma VNs were elucidated 11, 12. Recombinant human plasma VN and its recombinant domains bind to type I collagen under physiological conditions 13. The collagen‐binding activity of rat plasma VN is enhanced with decreased glycosylation during the liver regeneration induced by two‐thirds partial hepatectomy 14. Stepwise trimming of *N*‐glycans of VN with various exoglycosidases increases the collagen‐binding activity and induces formation of large‐sized multimer VNs 15.

Fibrosis of the liver, which synthesizes most VN, can progress to liver cirrhosis (LC). LC is a disease characterized by pathological accumulation and alteration of ECM proteins. The excessive accumulation and alteration of ECM proteins are led by continuous ECM remodeling during chronic liver injuries 16, 17. Collagens and VN are the main structural constituents of ECM. Therefore, the interaction between VN and collagen is considered to be the key to controlling ECM remodeling in LC, and increasing active VN against collagen would lead to advanced liver fibrosis, which results in cirrhosis. However, changes in glycosylation of VN and its collagen activity during LC have not been elucidated. If it is possible to control the modification of oligosaccharides on VN, it may be possible to open a way to retard progression of LC.

Carbon tetrachloride (CCl_4_) is toxic and induces liver lesions and liver fibrosis. Animals repeatedly administered CCl_4_ over time develop cirrhosis via fibrosis of the liver. Therefore, CCl_4_‐injected animals are commonly used as a model of chronic liver injuries such as LC. Rats treated with CCl_4_ for 8 weeks have a lipid profile, liver enzymes, and oxidative stress markers that are increased remarkably and total protein and high‐density lipoprotein levels that are decreased dramatically 18. However, the toxicological mechanisms of CCl_4_ remain not fully understood.

In this study, we focused on modulations of VN and determined the glycosylation and collagen‐binding activity of plasma VN in cirrhosis patients. We also examined the VN modulations in CCl_4_‐treated rats to evaluate the usefulness of rats with liver injury equivalent to chronic liver disease.

## Results

### Amount of total protein and VNs in human plasma

**Figure 1:**
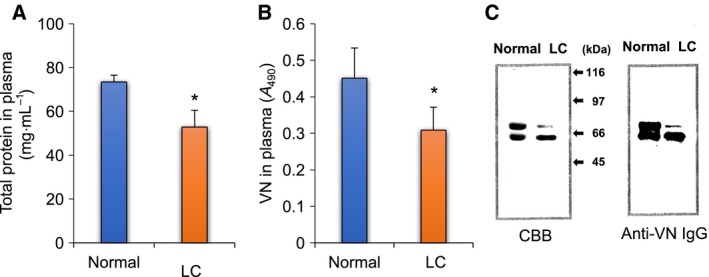
Protein and VN levels in LC plasma. (A) Total protein; plasma diluted 100–200 times was measured by using tunein‐TP. Normal: blue bar, LC: orange bar. (B) VN level in plasma; VNs in normal and LC plasma were measured by sandwich ELISA. Normal: blue bar; LC: orange bar. (C) SDS/PAGE and immunostaining of VNs; purified VNs (3 μg) from normal and LC plasma were loaded on each lane of 9.5% acrylamide gel, and SDS/PAGE was run in the presence of 2‐mercaptoethanol. Loading gels were transferred to PVDF membrane and stained with Coomassie brilliant blue (CBB) (left) or sheep anti‐VN IgGs and HRP‐anti‐sheep IgGs (right) as described in Materials and methods. Data are presented as mean ± SD. *P <* *0.05 compared to normal by Mann–Whitney *U* test. *n* = 10.

The total protein concentration in LC plasma decreased to approximately 72% of that in normal plasma (Fig. 1A). Parallel to the decrease of the total protein concentration in plasma, the VN level in LC plasma decreased to approximately 69% of that in normal plasma (Fig. 1B). VNs were purified from plasma samples by repeated heparin affinity chromatography before and after activation with urea treatment. The amounts of purified normal and LC VNs in 1 mL of plasma were approximately 0.12 and 0.04 mg, respectively. The yield of VNs purified from LC plasma was approximately 36% of that from normal plasma. Both normal and LC‐VNs gave double bands at the same migration positions of approximately 65 and 75 kDa on SDS/PAGE. The 75 kDa band of LC‐VN was thin compared with the 65 kDa band, while these two bands from normal VN had equivalent widths (Fig. 1C).

### Collagen binding activities of VNs

**Figure 2:**
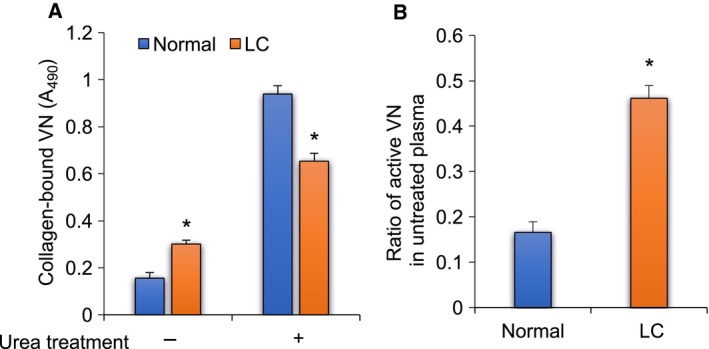
Binding activities of LC‐VNs toward collagen. (A) Collagen type I was immobilized in wells of 96‐well plates. Normal and LC plasma untreated or treated with 8 m urea were added to wells and incubated for 2 h at 37 °C. The binding studies were carried out by ELISA described as Materials and methods. The absorbance of collagen‐bound VNs was corrected using the reactivity of each VN to sheep anti‐VN IgGs. (B) The ratio of active VN in untreated plasma was obtained by dividing the collagen‐bound VNs in untreated plasma by the collagen‐bound VNs in urea‐treated plasma. Normal: blue bar; LC: orange bar. Data are presented as mean ± SD. *P <* *0.05 compared to normal by *t*‐test. *n* = 10.

**Figure 3:**
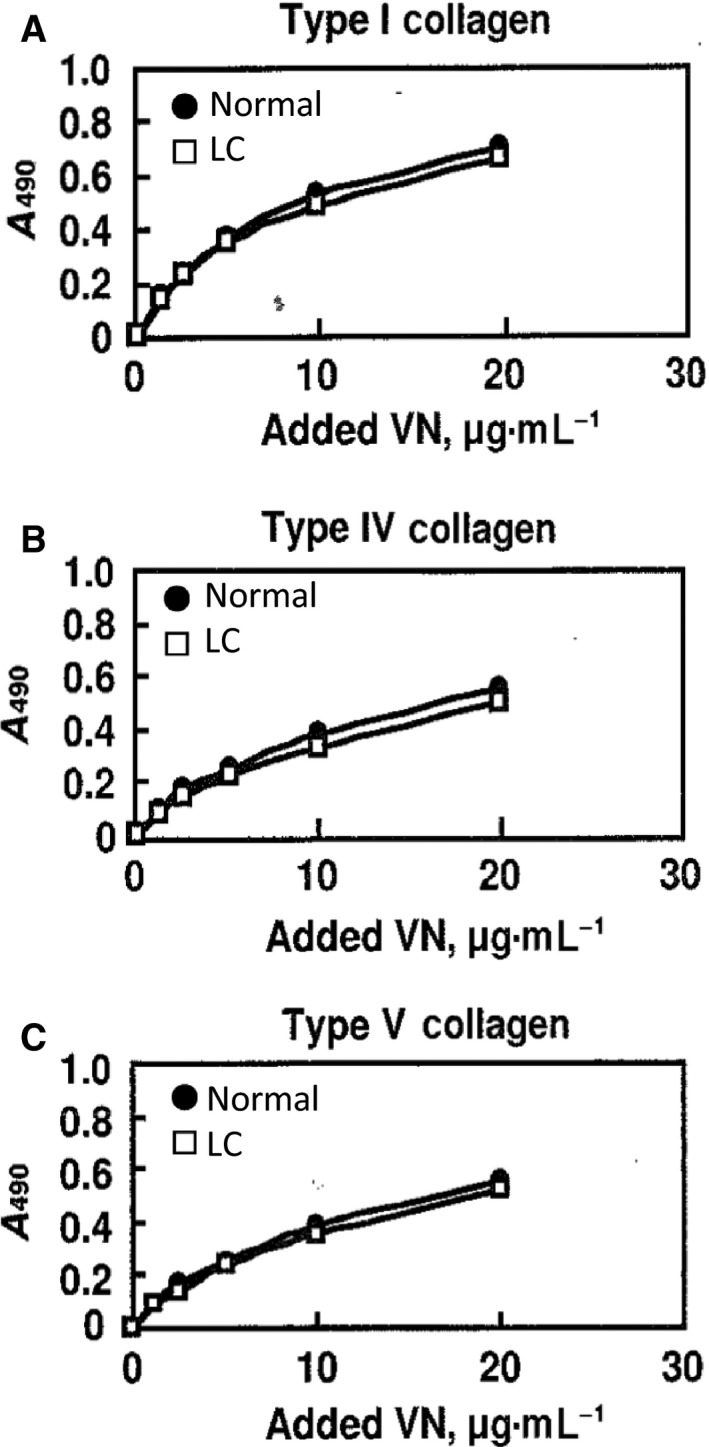
Collagen‐binding activities of purified LC‐VNs. Collagen type I (A), IV (B), and V (C) was coated on wells of a 96‐well plate. Purified normal or LC‐VN was added to wells and reacted with each collagen for 2 h at room temperature. Binding studies were carried out by ELISA as described in Materials and methods. The absorbance of collagen‐bound VNs was corrected using the reactivity of each VN to sheep anti‐human VN IgGs. Normal‐VN: solid circle; LC‐VN: open square.

Type I collagen‐binding activities of human plasma VNs were measured by ELISA. The inactive VN in human plasma was activated by urea treatment. The active VNs in normal and LC plasma before and after urea treatment were measured, and the ratio of active VN to total VN in plasma was calculated. In untreated plasma, VN in normal plasma showed low collagen‐binding activity compared with that in LC plasma, which suggests that the VN in normal plasma exists mainly in the native inactive form and the VN in LC plasma exhibits higher activity than that in normal plasma (Fig. 2A, urea treatment −). When natively inactive VNs in plasmas were activated by urea treatment, the collagen‐binding activities of the VNs in both normal and LC plasma increased in parallel with the VN levels in each plasma (Fig. 2A, urea treatment +). The ratio of active VN to total VN in untreated LC plasma was more than double that in normal plasma (Fig. 2B). The collagen‐binding activity of purified VNs was also measured by ELISA. Both normal and LC‐VNs dose‐dependently bound to all types of collagens, and the binding activity was in the following order: type I > V > IV. LC‐VN bound to all types of collagens like normal VN (Fig. 3A–C). These results showed that the ratio of active VN in LC plasma increased over that in normal plasma, while there was no difference between the purified normal and LC‐VNs in collagen binding.

### Carbohydrate analyses by HPLC and lectin reactivity

**Figure 4:**
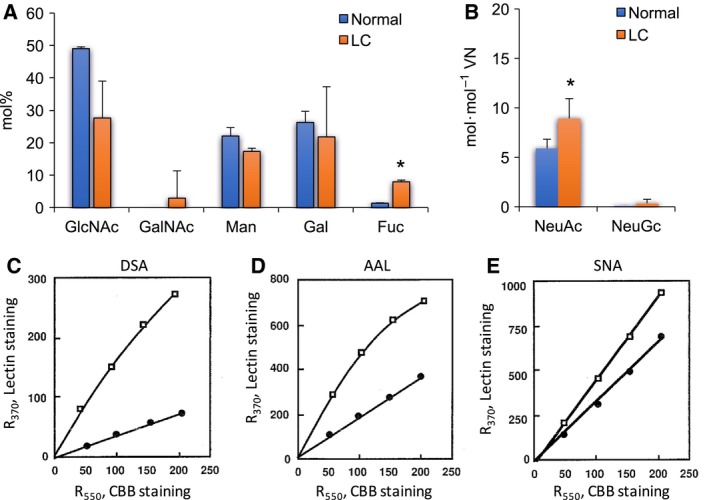
Carbohydrate analyses of LC‐VNs. (A,B) Carbohydrate analyses by HPLC. (A) Neutral sugars and hexosamines are expressed as mol% of total carbohydrates. Normal (blue bar) and LC (orange bar) VNs (4.5 μg) were hydrolyzed, *N*‐acetylated and labelled with 2‐aminopyridine (PA). PA‐carbohydrates were analyzed by reverse‐phase HPLC on a PALPAK Type A column as described in Materials and methods. (B) The sialic acid concentration is expressed as mol·mol^−1^ VN. After hydrolysis of normal (blue bar) and LC (orange bar) VNs (1.5 μg), released sialic acids were labelled with DMB and analyzed on a CLC‐ODS column as described in Materials and methods. (C–E) Dose dependency of lectin reactions. Various amounts of normal (closed circles) or LC (open squares) VN were dot‐blotted onto a PVDF membrane and stained with CBB or reacted with biotinyl lectins, DSA (C), AAL (D), and SNA (E). The staining intensities were measured by a refractive densitometer at 370 nm for lectin staining or 550 nm for CBB staining. Data are presented as mean ± SD. *P <* *0.05 compared to normal by *t*‐test. *n* = 3–4.

The carbohydrate composition of each purified VN was analyzed by HPLC after acid hydrolysis and fluorescence labelling. The noticeable changes in carbohydrates of purified VNs were increases in l‐fucose (Fuc) and *N*‐acetylneuraminic acid (NeuAc) in LC‐VN (Fig. 4A,B). Neutral sugars and hexosamines are expressed as mol% of total carbohydrates in each purified VN (Fig. 4A). These sugars, except for Fuc, did not change significantly, and the presence of d‐mannose (Man) and other neutral sugars suggested that both VNs contained complex‐type *N*‐glycans. There was no difference between normal VN and LC‐VN in total carbohydrates. NeuAc had increased in LC‐VN by 1.6‐fold that of normal VN (Fig. 4B). The lectin reactivities of VNs illustrate the changes in glycans during LC (Table 1). Both normal and LC VNs bound equally with ConA and *Ricinus communis* agglutinin (RCA). Enhanced reactivity of LC‐VN was observed for *Datura stramonium* agglutinin (DSA), *Aleuria aurantia* lectin (AAL), and *Sambucus nigra* bark agglutinin (SNA), which indicates increases of lactosamine branching, fucosylation to the innermost *N*‐acetylglucosamine (GlcNAc) residue, and sialyl α2‐6Gal sequences in LC‐VN, respectively, as shown in Fig. 4C–E. These results showed that the glycosylation of VN in the cirrhotic liver patients had changed dramatically from those of normal healthy individuals.

**Table 1:** Lectin reactivity of VNs. The lectin reactivity of VNs illustrates the partial structures of glycans. The reactivity of each VN was measured with biotinyl lectins on the membrane and expressed as staining intensity. +, positive staining; ++, +++, positive staining with strong intensity; −, negative staining; NT, not tested

Lectin	Human		Rat		Specificity
	Normal	LC	Control	CCl_4_	
Con A	++	++	++	+	N‐linked biantennary or oligomannose type
RCA	++	++	+++	+	Non‐reducing terminal Gal
DSA	+	++	+	++	Tri‐ or tetra‐antennary lactosamine type or (Galβ1‐4GlcNAc)_*n*_
LEL	−	−	+	++	Polylactosamine chain
AAL	+	++	+	+	Core or outer fucosyl residue
SNA	++	++	NT	NT	Sialylα2‐6 Gal
SNA*	−	−	NT	NT	
PVL	NT	NT	+	+++	Tri‐ or tetra‐antennary sialyl α2‐3 or non‐reducing terminal GlcNAc
PVL*	NT	NT	−	−	

* Reactivity after desialylation.

### VN from CCl_4_‐treated rats as a chronic liver disease model

It is difficult to obtain human disease samples for analyses of chronic liver disease. Therefore, the CCl_4_‐treated rat has often been used as a model of chronic liver disease. From the viewpoint of the functions of glycosylation of VN, CCl_4_‐treated rats were analyzed in this study.

**Figure 5:**
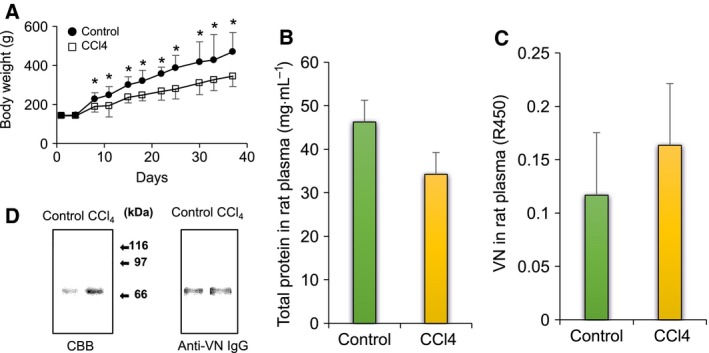
Amounts of protein and VN in CCl_4_‐treated rat plasma. CCl_4_ and olive oil were administered to rats twice a week. After 6 weeks, rats were sacrificed and blood collected. (A) Average weight of rats. Body weights of rats administered olive oil as a control (closed circle) and CCl_4_ (open square) were measured at the time of administration. *n* = 6–13. (B) Total protein. Diluted plasma (100–200 times) was measured by using tunein‐TP. Control: green bar; CCl_4_: yellow bar. *n* = 4. (C) VN level in plasma. Diluted plasma (2000–8000 times) was dot‐blotted onto a PVDF membrane and immunostained for VN. The staining intensity was measured by a refractive densitometer at 450 nm and corrected using the immunoreactivity of each VN. Control: green bar; CCl_4_: yellow bar. *n* = 4. (D) SDS/PAGE and immunostaining of VNs. Purified VNs (3 μg) from control and CCl_4_‐treated plasma were loaded on each lane of a 9.5% acrylamide gel, and SDS/PAGE was performed in the presence of 2‐mercaptoethanol. Loading gels were transferred to PVDF membranes and stained with CBB (left) or sheep anti‐VN IgG and HRP‐anti‐sheep IgGs (right) as described in Materials and methods. Data are presented as mean ± SD. *P <* *0.05 compared to control by Mann–Whitney *U* test.

The rats treated with CCl_4_ or olive oil as controls were weighed, and the concentrations of total plasma protein and the VN levels in plasma were measured. Liver injuries, including fibrosis and adherence to adjacent structures, were observed in CCl_4_‐treated rats (CCl_4_‐rats), and 4 out of 13 rats died, whereas the control rats were healthy. Blood biochemical parameters of liver damage [alanine aminotransferase (ALT) and aspartate aminotransferase (AST)] and liver function including parameters of cholestasis [total bilirubin (TBil)] in CCl_4_ plasma and LC plasma were measured to determine whether CCl_4_‐treated rats had induced liver injury corresponding to chronic liver disease. As shown in Table S1, ALT, AST, and TBil were increased to 300%, 480%, and 140% compared to controls, as in previous reports using CCl_4_‐treated rats as a chronic liver disease model 19, 20, 21, 22, 23. In addition, these parameter changes were similar to those of cirrhosis patients. The body weights of CCl_4_‐rats decreased with the repeated injections (Fig. 5A). VN levels in CCl_4_‐plasma increased compared with those in the control plasma while total protein concentrations decreased in CCl_4_ plasma (Fig. 5B,C). The yields of purified control and CCl_4_‐VN were approximately 21 and 34 μg·mL^−1^ of plasma, respectively. Both purified control and CCl_4_‐VNs gave a single band, at close migration positions of approximately 70 kDa in SDS/PAGE (Fig. 5D). These results of the VN of CCl_4_‐treated rats were the opposite of those of the VN of human LC in plasma VN concentrations and the amount of purified VN.

**Figure 6:**
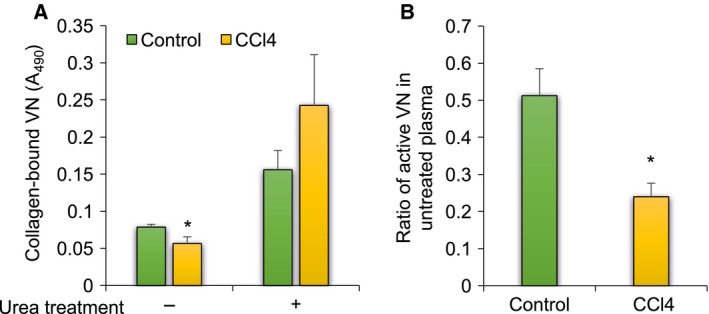
Collagen binding of VNs in CCl_4_‐treated plasma. (A) Collagen type I was immobilized on wells of a 96‐well plate. Control and CCl_4_‐plasma untreated or treated with 8 m urea were added to wells and incubated for 2 h at 37 °C. The binding studies were performed by ELISA as described in Materials and methods. The absorbance of collagen‐bound VNs was corrected using the reactivity of each VN to sheep anti‐VN IgGs. (B) The ratio of collagen‐binding VNs in the untreated plasma was calculated from the binding activities before and after urea treatment of plasma. Control: green bar; CCl_4_‐treated: yellow bar. Data are presented as mean ± SD. *P <* *0.05 compared to control by Mann–Whitney *U* test. *n* = 4–8.

Vitronectin in rat CCl_4_ plasma showed lower collagen‐binding activity compared with that in control rat plasma before urea treatment (Fig. 6A, urea treatment −). After urea treatment, the collagen‐binding activities of both VNs significantly increased (Fig. 6A, urea treatment +). The ratios of active VN in untreated plasma were decreased in the CCl_4_ plasma to approximately one‐half of that of the control plasma (Fig. 6B). These results suggest that the active VN in the CCl_4_ plasma was much lower than that in the control plasma. The results of CCl_4_‐treated rats were the opposite of those of human LC, too.

**Figure 7:**
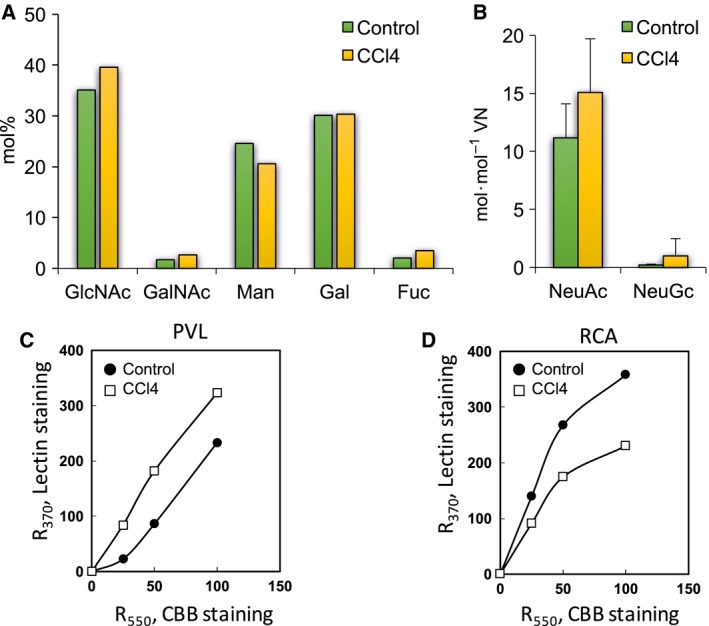
Carbohydrate analysis of CCl_4_‐VNs. (A,B) Carbohydrate analysis by HPLC. Carbohydrates in control (green bar) and CCl_4_‐treated (yellow bar) VNs (4.6 or 1.5 μg) were analyzed for neutral sugars or sialic acid concentrations, respectively, as described in Fig. 4A,B. (A) Neutral sugars and hexosamines are expressed as mol% of total carbohydrates. (B) Sialic acid concentration is expressed as mol·mol^−1^ VN. (C,D) Dose dependency of lectin reactions. Various amounts of control (closed circles) or CCl_4_ (open squares) VN was dot‐blotted onto PVDF membrane and stained with CBB or reacted with biotinyl lectins, PVL (C) and RCA (D). The staining intensities were measured by a refractive densitometer at 370 nm for lectin staining or 550 nm for CBB staining. Data are presented as mean ± SD.

In an HPLC analysis, mainly GlcNAc, galactose (Gal), and Man, as well as a minute amount of *N*‐acetylgalactosamine (GalNAc), were detected in control and CCl_4_‐VNs, suggesting that rat VNs contain mainly complex‐type *N*‐glycans and a few *O*‐glycans. In the composition of neutral sugars and hexosamines, there was no distinct difference between control and CCl_4_‐VNs (Fig. 7A). Amounts of NeuAc and NeuGc in CCl_4_‐VN tended to be higher than those in control VN (Fig. 7B). As shown in Table 1 and Fig. 7C, enhanced reactivity of CCl_4_‐VN was observed for *Psathyrella velutina* lectin (PVL), DSA, and *Lycopersicum esculentum* lectin (LEL), which indicates increases of sialyl α2‐3Gal sequences, lactosamine branching, and polylactosamine, respectively (Table 1 and Fig. 7C). Reduced reactivity of CCl_4_‐VN was observed for two *N‐*glycan‐specific lectins, ConA and RCA (Table 1 and Fig. 7D). Both rat VNs bound equally with fucose‐specific lectin, AAL (Table 1). These results suggested that complex type *N*‐glycans were reduced and that the sialic acid concentration tended to be increased in purified VN by CCl_4_. The changes in glycosylation of VN are different in CCl_4_‐treated rats and LC patients, except for NeuAc.

### Collagen‐binding activities of purified VNs treated with glycosidases

**Figure 8:**
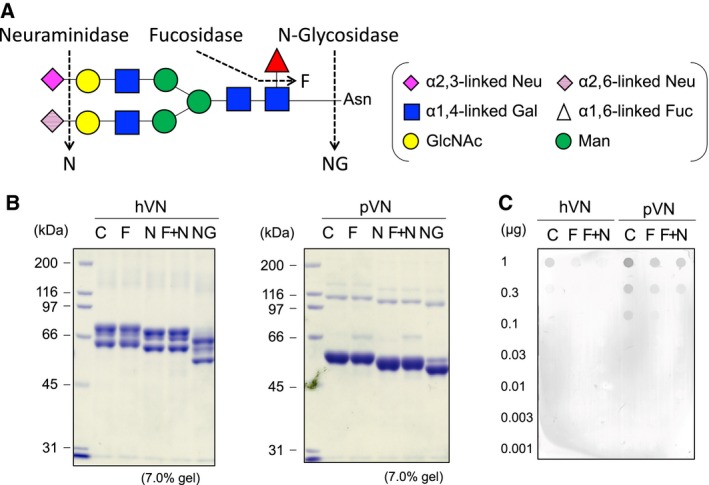
Preparation of hVN and pVN treated with glycosidases. (A) Cleavage site of enzymatic deglycosylation in the structure of the fucosylated *N*‐glycan of hVN and pVN. (B) Glycosidase‐treated hVNs and pVNs (4 μg per lane) were loaded in each lane of 7.0% polyacrylamide gel and run for SDS/PAGE under reducing conditions with 2‐mercaptoethanol. The gels were stained with CBB. (C) Dot blot analysis of PVDF membrane stained with the biotinylated UEA‐I for detecting Fuc in VNs. C, control VN incubated without enzyme; F, VN treated with fucosidase; N, VN treated with neuraminidase; F+N, VN treated with both fucosidase and neuraminidase; NG, VN treated with N‐glycosidaseF.

**Figure 9:**
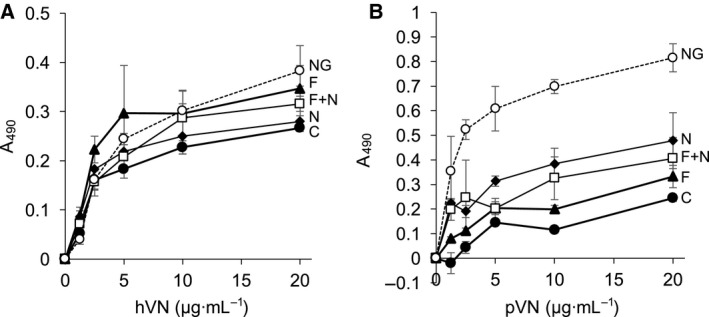
Collagen‐binding activities of glycosidase‐treated VNs. Collagen type I was immobilized on wells of a 96‐well plate. The purified glycosidase‐treated VNs were added to the wells and reacted with the collagen in 10 mm Tris/HCl saline (pH 7.5). The VNs bound to collagen were detected by rabbit anti‐VN IgGs (1/25 000, LSL‐LB‐2096; Cosmo Bio Co., Ltd) and goat anti‐rabbit IgGs Fc‐HRP (1/10 000, AP156P; Millipore Corp.). Binding studies were carried by ELISA as described in Materials and methods. The absorbance of collagen‐bound VNs was corrected using the reactivity of each VN to anti‐VN IgGs. C, control VN incubated without enzyme (●); F, VN treated with fucosidase (▲); N, VN treated with neuraminidase (♦); F+N, VN treated with both fucosidase and neuraminidase (□); NG, VN treated with N‐glycosidaseF (○). (A) hVN, (B) pVN. Data are presented as mean ± SD.

Collagen‐binding activities of VNs treated with glycosidases were measured to clarify the relationship between carbohydrate and collagen‐binding activities of purified LC‐VN. Human VN (hVN) and porcine VN (pVN) were used for analysis because hVN has only a few *N*‐glycans with Fuc, but pVN has many *N*‐glycans with Fuc (Table 2) 11, 12. hVN (65 and 75 kDa) and pVN (58 kDa) were purified from human and porcine plasma, and carbohydrates on the VNs were trimmed by fucosidase (F), neuraminidase (N), or a combination of F and N and N‐glycosidaseF (NG) (Fig. 8A). de*N*‐glycosylation by NG and desialylation by N were confirmed by the lower molecular masses of VNs (Fig. 8B). Because defucosylation by F could not be confirmed by molecular mass reduction, it was confirmed by staining reduction of the Fuc‐recognition lectin *Ulex europaeus* agglutinin (UEA‐I) in dot‐blot analyses (Fig. 8C). Binding of VNs treated with glycosidases to collagen type I showed the same tendency in hVN and pVN, and the binding increased in the following order: NG > F, N, F + N > C (Fig. 9). These results suggest that defucoslylation and desialylation of purified VNs slightly increased the collagen‐binding activity and that the collagen‐binding activity was most enhanced by de*N*‐glycosylation of purified VNs.

**Table 2:** Molar ratio and structure of N‐linked oligosaccharides on hVN and pVN 11, 12

hVN			pVN		
Fraction	Molar ratio (%)	Structure	Fraction	Molar ratio (%)	Structure
hM‐1	3.4	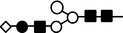	pM‐1	3.0	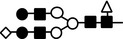
hM‐2	2.7	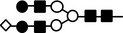	pM‐2	1.7	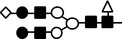
hM‐3	1.0	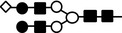	pD‐3	7.6	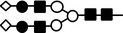
hD‐2	45.8	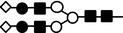	pD‐4	51.8	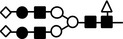
hD‐3	3.1	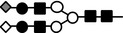	pD‐5	2.2	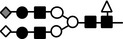
hD‐4	7.9	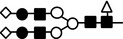	pT‐1	2.3	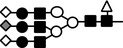
hT‐2	9.0	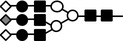	pT‐2	2.1	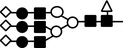
hT‐3	3.8	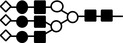			

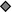
: α2, 3‐linked Neu; 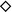: α2, 6‐linked Neu; 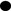: β1, 4‐linked Gal; 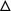: α1, 6‐linked Fuc; 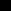: GlcNAc; 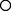: Man.

## Discussion

This study showed that human VN in cirrhotic patients differs from VN in normal individuals in the plasma VN level, the ratio of active VN, and its glycosylation (Figs. 1, 2, 4). The VN synthesis appears to parallel the decrease in protein synthesis caused by lowered liver function due to cirrhosis (Fig. 1). Under the untreated physiological condition, a higher percentage of VN was found to be the active form in LC plasma than in normal plasma (Fig. 2A, urea treatment − and B). These results suggest that the increase of active VN in cirrhotic plasma may contribute to the incorporation of plasma VN into the matrix and subsequent repair or remodeling processes during cirrhosis. On the other hand, the amount of VNs activated by treatment with urea was lower in LC plasma than in normal plasma (Fig. 2A, urea treatment +). Because urea activates inactive VN, the amount of VN treated with urea corresponds to the total amount of VN in plasma (Fig. 1B).

In this study, two‐step heparin‐affinity chromatography was used for purification of VN 8. In the first step, originally active VN in plasma binds to a heparin column under a non‐denaturing condition and is removed. The inactive VN in the pass‐through fraction in the first step is activated by urea and purified by the second step. The purified VNs were originally inactive and artificially activated forms. Therefore, the yield of purified LC‐VN was approximately one‐third of normal VN, as seen in Fig. 2A.

**Figure 10:**
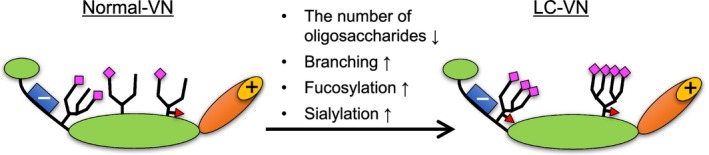
Schematic representation of the changes of *N*‐glycan on LC‐VN expected from these results. The number of oligosaccharides transferred to the VN peptide decreased, while branching, fucosylation, and sialylation of oligosaccharides in the LC‐VN increased compared to normal VN.

In the carbohydrate analyses of purified VNs, the mol% values of Fuc and NeuAc were increased, while those of GlcNAc, Gal, and Man tended to decrease (Fig. 4). The reactivities with DSA, AAL, and SNA were enhanced in LC‐VN (Table 1). The results suggest that the number of oligosaccharides transferred to the VN peptide decreased, while branching, fucosylation, and sialylation of oligosaccharides in LC‐VN increased. Figure 10 is a schematic representation of the changes of *N*‐glycans in LC‐VN expected from these results. Collagen‐binding activities of both hVN and pVN treated with glycosidases were in the order NG > F, N, F + N > C (Fig. 9). Among the changes in glycosylation of LC‐VN, the number of oligosaccharides transferred to the VN peptide decreased, which would enhance collagen‐binding activity of LC‐VN because the collagen‐binding activities of VNs were enhanced by de*N*‐glycosylation, as shown in Fig. 9. On the other hand, changes in the glycosylation of LC‐VN, branching, fucosylation, and sialylation of oligosaccharides would reduce the collagen‐binding activity of LC‐VN because collagen‐binding activity of VNs was enhanced by defucosylation and desialylation, as shown in Fig. 9. Consequently, the purified human VNs from normal and LC plasma showed almost the same binding to collagens (Fig. 3), which can be considered to be a result of preventing the enhancement due to the decreasing *N*‐glycosylation and the suppression due to the increase of Fuc and sialic acids. Previously, we showed that the collagen‐binding activity of purified VN is enhanced by the change in glycosylation *in vitro* and during liver regeneration after partial hepatectomy *in vivo* 14. In glycosylation changes, carbohydrate composition analysis and lectin reactivity indicated a decrease in sialic acid in VN from partially hepatectomized rats at 24 h. The desialylation and de*N*‐glycosylation of purified rat VN increased the collagen‐binding activity by 2.9 and 1.2 times, respectively 14. The desialylation and de*N*‐glycosylation of human VN also increased the collagen‐binding activity by 1.5 and 2.8 times, respectively 15. This study clarified not only the reproducibility of these previous reports but also the effect of Fuc in carbohydrates on VN in collagen‐binding activity, as shown in Figs 8 and 9.

In LC, significantly high levels of serum sialic acid were reported as compared to controls 24. The levels of fucosylated species of α‐fetoprotein in patients with hepatocellular carcinoma, LC, and chronic hepatitis are increased compared with those in normal controls 25. The plasma fucosyltransferases are specifically elevated in hepatocellular carcinoma 26. The changes of VN glycosylation observed in LC (Fig. 4) agree with the reported changes of other serum proteins and related enzymes such as glycosyltransferases. In this study, we clarified that the oligosaccharide structures of the purified LC‐VN, which were originally inactive, were activated by urea treatment during the purification step. Therefore, in future work, it will be necessary to clarify the oligosaccharide structures on active VN in order to elucidate the relationship between oligosaccharide structures on VN and the collagen‐binding activity in LC in more detail. However, for this future subject, a method for isolating active VN from plasma has not been established. Thus, this problem needs to be solved before such future studies.

Plasma samples of human patients are limited. Therefore, we attempted to determine whether CCl_4_‐treated rats are an appropriate model of LC to study the mechanism of the pathological matrix remodeling in view of VN. Several effects of CCl_4_ injection into rats were observed on the plasma VN level, the ratio of active forms, and the glycosylation of VN when compared to the rats injected with olive oil as a control. However, the effects were almost the opposite of those of human cirrhotic disease except for a small part of the glycosylation changes (Figs 5, 6, 7). Rat VN is both *N*‐ and *O*‐glycosylated, and the molecular mass is uniformly 70 kDa. Human VN is only *N*‐glycosylated and exists in two forms, with the molecular mass of 75 kDa and the nicked products of 65 kDa plus 10 kDa 11, 12. The amino acid sequence identity between human and rat VNs is 73% with four common domains and ligand‐binding activities. Human VN has three *N*‐glycosylation sites (Asn 86, 169, and 242), all of which are conserved in rat VN with one additional *N*‐glycosylation site (Asn 96), but rat VN is also *O*‐glycosylated. The results of this study clarify that the CCl_4_‐treated rat has a significantly different pathology from human LC when VN is the focus.

In summary, we showed that the VN level in plasma was decreased but active VNs were increased in chronic liver disease. The glycosylation of VN was also changed, with high fucosylation and sialylation in chronic liver disease. In contrast, VN levels in plasma were increased but active VN was decreased in CCl_4_‐treated rats, while glycosylation changes were observed in CCl_4_‐VN. These data suggested two findings: one is that the glycosylation of VN might modulate the collagen‐binding activity related to liver fibrosis in chronic liver disease, and the other is that CCl_4_‐treated rats are not an appropriate model for the VNs in human LC disease.

## Materials and methods

### Materials

Sheep anti‐human VN IgGs were purchased from the Binding Site Ltd (Birmingham, UK), and horseradish peroxidase (HRP)‐conjugated rabbit anti‐sheep IgGs were purchased from ICN Biomedicals, Inc. (Costa Mesa, CA, USA). Rabbit anti‐VN IgGs were purchased from the Cosmo Bio Co., Ltd (Tokyo, Japan), and HRP‐conjugated goat anti‐rabbit IgG was purchased from Millipore (Temecula, CA, USA). Various lectins, concanavalin A (ConA), *R. communis* agglutinin (RCA), *D. stramonium* agglutinin (DSA), lectins of *L. esculentum* (LEL) and *A. aurantia* (AAL), *Sambucus nigra* bark agglutinin (SNA), and *Ulex europaeus* agglutinin (UEA‐I) were purchased from Seikagaku Kogyo (Tokyo, Japan). *P. velutina* lectin (PVL) was purified from the fruiting bodies of *P. velutina* mushrooms collected in Japan 1, 2. Lectins were labeled with *N*‐hydroxysuccinimide biotin (Pierce, Rockford, IL, USA) according to the method reported previously 2. Collagen types I, IV, and V from porcine skin and other reagents of special grade were from Wako Pure Chemicals (Osaka, Japan). Fucosidase (GKX‐5006) from bovine kidney was purchased from Prozyme (Hayward, CA, USA). Neuraminidase (sialidase) from *Arthrobacter ureafaciens* and N‐glycosidase F from *Flavobacterium meningosepticum* and expressed in *Escherichia coli* were purchased from Roche Diagnostics (Mannheim, Germany).

### Plasma samples

Human plasma samples from 10 healthy normal people were provided by I. Ishizuka of Teikyo University and S. Yamada of Tottori University. Human plasma samples from 10 LC patients were provided by S. Yamada of Tottori University and S. Oda‐Tamai of St Marianna University. Informed written consent was obtained from all subjects in accordance with the Ethical Guidelines for Medical and Health Research Involving Human Subjects published by the Ministry of Health, Labor and Welfare, Japan (MHLW). This study was approved by the Research Ethics Committee of Ochanomizu University and conforms to the standards set by the Declaration of Helsinki (2018‐2). Male Wistar rats aged 8 weeks (weighing 130–150 g; Nihon Clea, Tokyo, Japan) were maintained at a constant temperature (23.5 °C) with 12 h each light (06.00–18.00 h) and darkness. Control or CCl_4_‐group rats were injected intraperitoneally with olive oil (1 mL·kg^−1^) or CCl_4_ (2 mL·kg^−1^, CCl_4 _: olive oil = 1 : 1), respectively, twice a week for 6 weeks. Rats were anesthetized and sacrificed, and blood was collected. Anticoagulant sodium citrate 3.8% (w/v) solution was added to blood at a ratio of 1 : 10 (v/v). Animal experimental procedure and housing condition were approved by the Institutional Animal Care and Use Committee of Ochanomizu University. Porcine plasma was purchased from Tokyo Shibaurazouki Co., Ltd (Tokyo, Japan).

### Purification of VN from plasma

Vitronectins were purified from each plasma by repeated heparin‐affinity column chromatography before and after urea treatment according to the methods of Yatohgo *et al*. 8. Heparin–Sepharose, which is stable and has a high‐binding capacity for VN, was prepared by reductive amination 29. VN was eluted with 0.5 m NaCl from the heparin column (1.8 × 4 cm, 10 g gel) in a urea‐denatured condition and then refolded by step‐wise dialysis with the ion concentration decreasing to 0.13 m from 2 m NaCl.

### Protein determination

Total protein in plasma was measured by using a protein assay kit, Tunein‐TP (Otsuka Pharmaceutical Co., Ltd., Tokyo, Japan). The plasma sample was diluted 100–200 times (5 μL) and mixed with the protein assay reagent (300 μL) in 96‐well Iwaki assay plates. After standing for 5 min at room temperature, the absorbance of the sample was measured at 595 nm using a microplate reader (Vient, DS Pharma Biomedical, Osaka, Japan). Bovine serum albumin was used as a protein standard.

### Quantification of VNs in plasma

The VN concentration of plasma was measured by sandwich ELISA or dot‐blotting using an anti‐VN polyclonal antibody (sheep anti‐human VN IgGs) and labelled polyclonal antibody. For ELISA, sheep anti‐human VN IgGs (0.2 μg·mL^−1^, 100 μL) were immobilized in wells of 96‐well plates (Immulon 1; Dynatech Laboratories, Alexandria, VA, USA) at 4 °C overnight. Then wells were blocked with 5% BSA in 10 mm PBS (pH 7.5) for 2 h at room temperature. Each plasma sample was diluted with PBS to 100–5000 times (50 μL) and added to each well, followed by incubation at room temperature for 1 h. The amount of VN in each well was determined by biotin‐labeled sheep anti‐human VN IgGs (0.2 μg·mL^−1^, 100 μL) and then HRP–avidin–biotin complex (4 μg·mL^−1^, 100 μL) at room temperature for 1 h each. After washing with PBS four times, the bound HRP was developed using 0.04% *O*‐phenylenediamine in 0.1 m phosphate–citrate buffer (pH 5.0) containing 0.01% H_2_O_2_ (200 μL), stopped by addition of 2 m H_2_SO_4_ (50 μL), and measured at 490 nm using a microplate reader (model 680, Bio‐Rad, Hercules, CA, USA). For dot‐blotting, two‐fold dilution series of plasma samples (each 100 μL) were dot‐blotted onto polyvinylidene fluoride (PVDF) membranes and blocked with 3% BSA, then cut into lanes and reacted with the biotin‐labeled sheep anti‐human VN IgGs (1 μg·mL^−1^) and the HRP–avidin–biotin complex (1 μg·mL^−1^) at room temperature for 2 h each. The membrane was developed with 0.05% 4‐chloro‐1‐naphthol containing 0.01% H_2_O_2_. The staining intensities were measured by a refractive densitometer (Shimadzu CS9300PC, Shimadzu, Kyoto, Japan).

### SDS/PAGE and immunoreactivity of purified VNs

Vitronectins were loaded on each lane of a 9.5% polyacrylamide gel, and SDS/PAGE was run in the presence of 2‐mercaptoethanol according to the methods of Laemmli 30. Proteins were blotted onto a PVDF membrane and stained with Coomassie brilliant blue (CBB) or immunostained with sheep anti‐human VN IgGs and HRP‐conjugated rabbit anti‐sheep IgGs. The color was developed with 0.05% 4‐chloro‐1‐naphthol containing 0.01% H_2_O_2_.

### ELISA for binding of VNs to collagen

Collagen‐binding activities were assayed by ELISA. Collagen types I, IV, and V (10 μg·mL^−1^ in 0.1 m carbonate buffer, pH 9.5, 100 μL) were immobilized onto wells of a 96‐well Immulon 1 plate for 3 h at room temperature, and then the wells were blocked with 300 μL of 0.5% skim milk in PBS overnight at 4 °C. Purified VN or plasma before and after urea treatment (50 μL) was added to each well, followed by incubation for 2 h at room temperature. The VNs bound to the immobilized collagens were detected with sheep anti‐human VN IgG and HRP‐conjugated rabbit anti‐sheep IgG. After washing with PBS (300 μL) four times, the HRP was developed by the same method described for sandwich ELISA in the Materials and methods. For activation of plasma VNs by urea treatment, urea was added to the plasma, then the volume was adjusted to 10 volumes with PBS containing 5 mm EDTA and 1 mm phenylmethylsulfonyl fluoride to a final concentration of 8 m urea 31. The solution was incubated for 2 h at 37 °C and diluted with 8 volumes of PBS for ELISA.

### Carbohydrate analyses by HPLC

Vitronectins were dot‐blotted onto the PVDF membranes and washed with Milli‐Q (Merck Millipore, Burlington, MA, USA) water. The membranes were placed in glass tubes and dried in a vacuum desiccator over KOH. Then hydrolysis was carried out *in vacuo* with a vapor of 2 m HCl and 2 m trifluoroacetic acid for 4 h at 100 °C. After hydrolysis, the released sugars in the mixture were *N*‐acetylated and labelled with a fluorescent probe, 2‐aminopyridine (PA), as described previously 14, and PA‐carbohydrates were analyzed by reverse‐phase HPLC on a PALPAK Type A column (4.6 × 150 mm; Takara, Shiga, Japan) according to the method previously reported 14, 32. The sialic acid component was analyzed according to the method of Hara *et al*. 33. VNs (1.5 μg) were hydrolyzed with 0.025 m HCl at 80 °C for 1 h, and the sialic acids released were labelled with a fluorescent probe, 12‐diamino‐4,5‐methylenedioxy‐benzene, dihydrochloride‐2 HCl containing 1.0 m β‐mercaptoethanol and 10 mm Na_2_S_2_O_4_, and they were analyzed on a Shim‐pack CLC‐ODS column (6.0 × 150 mm; Shimadzu).

### Reactivity of VNs with biotin‐lectins

Two‐fold dilution series of VN samples (each 100 μL) were dot‐blotted onto PVDF membranes and blocked with 3% BSA, then cut into lanes and reacted with each biotinyl lectin in PBS 14. After reacting with HRP–avidin–biotin complex, the membrane was developed with 0.05% 4‐chloro‐1‐naphthol containing 0.01% H_2_O_2_ according to the method previously reported 14. The staining intensities were measured by a refractive densitometer (Shimadzu CS9300PC), at an absorbance of 370 nm for lectin staining or at 550 nm for CBB staining. Asialoglycoproteins were prepared by desialylation of intact glycoproteins by the treatment with 0.01 m HCl at 80 °C for 1 h.

### Correction for VN detection by relative antibody reactivity or protein of VNs

Vitronectins were dot‐blotted onto PVDF membrane and reacted with sheep anti‐human VN IgG and HRP‐conjugated rabbit anti‐sheep IgG. The membrane was developed with 0.05% 4‐chloro‐1‐naphthol containing 0.01% H_2_O_2_. The raw absorbance of 490 nm data of bound VN in ELISA was corrected using the relative antibody reactivity of each VN because the immunoreactivity against VN from CCl_4_ plasma was higher than that of control plasma.

### Liver biochemical tests

All blood samples were stored at −20 °C until use. Concentrations of ALT and AST and liver function tests including parameters of cholestasis (TBil) were determined spectrophotometrically using an automatic biochemical analyzer by Oriental Yeast Co., Ltd (Shiga, Japan).

### Glycosidase digestion of VN

Enzyme deglycosylation of hVN and pVN was performed according to the manufacturer's instructions (Roche Diagnostics, Indianapolis, IN, USA) and as described previously 14, 15, 34, 35. VNs (150 μg) were treated with fucosidase (50 mU), neuraminidase (5 mU), fucosidase and neuraminidase (50 and 5 mU, respectively), or N‐glycosidaseF (1 U) in 20 mm sodium citrate phosphate buffer (pH 6.0) at 37 °C for 48 h. The control was prepared by adding the same volume of the buffer as the enzymes. The treated VNs were dialyzed against PBS.

### Dot blot analysis

Three‐fold dilution series of glycosidase‐treated VNs (each 100 μL) were dot‐blotted onto PVDF membranes and blocked with 3% BSA in 10 mm Tris/HCl saline (pH 7.5), then cut into lanes. The spots were reacted with the biotinylated UEA‐I, which specifically binds to Fuc, and subsequently with HRP–avidin–biotin complex (diluted 1 : 1000) at room temperature for 1 h each. The spots containing Fuc were detected using ECL reagent (GE Healthcare GE Helthcare (Little Chalfont, Buckinghamshire, UK) ECL^TM^ Western Blotting Detection Reagents), and the intensity of each spot was visualized with an ImageQuant LAS4000 mini imager (GE Healthcare).

### Statistical analysis

Data are expressed as the mean ± standard deviation (mean ± SD) and compared by the non‐parametric Mann–Whitney *U* test or *t*‐test. A *P* value of < 0.05 was assigned significance. All statistical analyses were done with ibm spss statistics (version 23; IBM Corp., Armonk, NY, USA).

## Conflict of interest

The authors declare no conflict of interest.

## Author contributions

KD and RS performed the experiments and analyzed the data. KD and HO wrote the manuscript. HO conceived the study. SO‐T and HO initiated the study. All authors reviewed the manuscript.

## Supporting information


**Table S1.** Comparison of this study and previous reports using a rat liver cirrhosis model. Conditions of CCl_4_ administration and alteration of body weights and blood parameters after CCl_4_ administration. NT, not‐tested. Data are presented as mean ± SD. *P <* *0.05, comparison of CCl_4_ to control by Mann–Whitney *U* test. *n* = 4.
