# Supplementary material for: Vitronectins produced by human cirrhotic liver and CCl4‐treated rats differ in their glycosylation pattern and tissue remodeling activity
Source: FEBS Open Bio. 2019 Mar 18;9(4):755–68. doi: 10.1002/2211-5463.12616 (PMC6443879; doi:10.1002/2211-5463.12616)
Supplement: Supplementary file 1 — Table S1. Comparison of this study and previous reports using a rat liver cirrhosis model. Conditions of CCl4 administration and alteration of body weights and blood parameters after CCl4 administration. NT, not‐tested. Data are presented as mean ± SD. *P < 0.05, comparison of CCl4 to control by Mann–Whitney U test. n = 4. [file FEB4-9-755-s001.doc]

**Supplementary Table 1. Comparison of this study and previous reports using a rat liver cirrhosis model.** Conditions of CCl_4_ administration and alteration of body weights and blood parameters after the CCl_4_ administration. NT, Not-tested. ^*^, *p* < 0.05 compared CCl_4_ to control by Mann-Whitney U test. n=4.

|  | Conditions of CCl_4_ administration | | | | Changes after CCl_4_ administration | | | |
| --- | --- | --- | --- | --- | --- | --- | --- | --- |
|  | Periods | Concentration | Volume | Times /week | Body weight  (% of Control) | Blood biochemical parameters  (% of Control) | | |
|  |  |  |  |  |  | ALT | AST | TBil |
| This Study | 6 weeks | 50% CCl_4_ | 2 ml/kg | Two | 73% | 300%^*^ | 480%^*^ | 140%^*^ |
| Ref. 1 [19] | 12 weeks | 50% CCl_4_ | 0.20-0.45 ml/kg | Two | 65% | 280% | 390% | 450% |
| Ref. 2 [20] | 12 weeks | 5% CCl_4_ | 5 ml/kg | Two | 92% | 500% | 350% | 220% |
| Ref. 3 [21] | 6 weeks | 50% CCl_4_ | 2 ml/kg | Two | NT | 270% | 180% | NT |
| Ref. 4 [22] | 6 weeks | 25% CCl_4_ | 1 ml/kg | Two | 84.43% | NT | NT | NT |
| Ref. 5 [23] | 6 weeks | 50% CCl_4_ | 1 ml/kg | Two | NT | 340% | 400% | NT |
| This Study | Cirrhosis patients | | | | NT | 130% | 140% | 110% |
